# Partnerships, Collaborations, and Investments Integral to CDC’s International Response to COVID-19

**DOI:** 10.3201/eid2813.221751

**Published:** 2022-12

**Authors:** Rochelle P. Walensky

**Affiliations:** Centers for Disease Control and Prevention and Agency for Toxic Substances and Disease Registry, Atlanta, Georgia, USA

**Keywords:** COVID-19, infectious disease outbreaks, global health security, US President’s Emergency Plan for AIDS Relief, PEPFAR, coronavirus disease, SARS-CoV-2, severe acute respiratory syndrome coronavirus 2, viruses, respiratory infections, vaccine-preventable diseases

Since SARS-CoV-2 was first identified, the world has witnessed more than 641 million confirmed cases of COVID-19, resulting in more than 6.6 million deaths (*1*). The global spread of the virus and the resulting destruction of lives and livelihoods brought into sharp focus the interconnectedness of local, domestic, and global public health infrastructure and the global need for a trusted, resilient public health workforce to overcome systemic inequities.

As the public health agency for the United States, the Centers for Disease Control and Prevention (CDC) invests in global and domestic public health to improve core public health capabilities. CDC collaborates with partners in the interdependent global public health ecosystem to strengthen the systems needed for disease surveillance and reporting, diagnostic testing, outbreak and pandemic responses, and clinical service delivery, including treatment, immunizations, and infection prevention and control.

Internationally, CDC staff work side-by-side with the staff of ministries of health and other public health institutions in more than 60 countries, providing technical guidance, training the next generation of disease detectives and public health emergency responders, and addressing both global and local public health challenges. Recognizing historic power imbalances that continue today, together we are building, modernizing, and bolstering health programs and developing integrated, functional, and flexible public health systems that are country-owned and sustainable. This technical assistance is driven by science and data and is designed to address the unique needs of each country. Support did not begin with the arrival of SARS-CoV-2; rather, these alliances date back many decades.

To help strengthen a stronger path to the future, it is important to recognize the role these long-standing partnerships and investments in country infrastructure played when SARS-CoV-2 arrived. This infrastructure included (to name a few) facility-based testing, treatment, and prevention services; surveillance and laboratory systems; workforce and institutional development; and emergency preparedness infrastructures developed through the US President’s Emergency Plan for AIDS Relief (PEPFAR) since 2003 (*2*). Taken together and coupled with the implementation of the Global Health Security Agenda in 2014, countries have strengthened capacities to prevent, detect, and respond to public health threats (*3*). In many countries, laboratory systems supported through PEPFAR and global health security investments facilitated rapid roll-out of SARS CoV-2 diagnostic testing (*4*,*5*). This supplement issue of the Emerging Infectious Diseases journal highlights these foundational health systems, programs, and platforms that not only continued to support the public health challenges upon which they were built, but swiftly adapted to the complexities of COVID-19 (*6*).

In partnership with CDC, some countries drew on public health workforce and institutional development programs to respond to COVID-19. By July 2021, a total of 32 Field Epidemiology Training Programs (FETPs), CDC’s flagship program for training a global workforce of field epidemiologists, engaged nearly 10,000 FETP residents and graduates to support global COVID-19 epidemiologic investigations, data collection and analysis, and information dissemination (*7*). In addition, the Stop Transmission of Polio (STOP) Program, a collaboration between CDC, the World Health Organization, and the United Nations Children’s Fund (UNICEF) that has recruited, trained, and deployed international public health professionals since 1998 to strengthen national immunization systems for polio eradication and the control and prevention of all vaccine-preventable diseases, also supported COVID-19 response activities (*8*).

During recent visits to Tanzania and Uganda, I saw firsthand how these collaborations and investments were leveraged to benefit COVID-19 response activities. In Tanzania, where CDC has enjoyed a 2-decade long collaboration with the Ministry of Health, HIV treatment facilities and local partners provided COVID-19 vaccines to clients during appointments for antiretroviral therapy. HIV clinics were dually purposed to provide COVID-19 vaccines and to train on-site staff in their administration; more than 1,000 of these vaccine stations were supported by CDC. Through these efforts and broader vaccination campaigns and community outreach to underserved communities, Tanzania vaccinated millions of people for COVID-19 (*9*).

In Uganda, more than 3 decades of partnership and national public health progress against HIV and other infectious diseases built the foundation for quick action and early successes during the COVID-19 response (for example, the laboratory network for HIV and TB diagnostics developed through PEPFAR was used for COVID-19 testing and specimen transport) (*10*). Uganda FETP fellows and graduates supported all aspects of the COVID-19 response, including conducting contact tracing and case surveillance (*11*). Those assets and capacities were essential for rapid response to COVID-19 and continue to be used for other emerging and reemerging infectious disease outbreaks, including the most recent Ebola outbreak (*7*,*10*–*12*).

Vietnam’s work to develop national guidelines, strengthen laboratory testing, and provide infection prevention and control training to hospital staff (*13*); Thailand’s COVID-19 testing in refugee camps and work to strengthen border health activities and point-of-entry assessments (*14*); Brazil’s investigation of the second wave of COVID-19 and the P.1 and B.1.162 variants (*15*); and Ukraine’s implementation of a COVID-19 mitigation strategy for a 2021 religious pilgrimage that drew tens of thousands of pilgrims to the city of Uman (*16*) were all enhanced through longstanding CDC partnerships. In 2020, a total of 41 PEPFAR-supported countries had overall gains in HIV treatment and viral load suppression because of innovations and adaptations in HIV service delivery (*17*) implemented in the context of the COVID-19 pandemic, which also were made possible because of collaborations with CDC.

In the pandemic’s aftermath, decades of global progress against vaccine-preventable diseases remains threatened. From 2019 to 2021, the number of unvaccinated and under-vaccinated children around the world increased from 19 to 25 million, the highest number recorded since 2008, and the number of zero-dose children (those completely unvaccinated against diphtheria, tetanus, and pertussis) substantially increased, from 13 to 18 million (*18*). Global efforts to recover from these setbacks are focused on bolstering national immunization programs to reach every child through catch-up vaccinations for polio, measles, and other vaccine-preventable diseases. Other efforts include capitalizing on COVID-19 vaccination rollouts to strengthen essential immunization programs.

We must continuously build and invest in public health capacity in the United States and globally to protect our nation and the world against dangerous and costly health threats so that we are well-positioned to swiftly respond when and where those threats arise. We also need to strategically increase surveillance and laboratory capacity for existing outbreak-prone and new emerging pathogens, constantly assess and strengthen partnerships, support equitable access to medical countermeasures, and regularly evaluate indicators that measure progress. CDC’s science needs to be proactively shared with the public in an understandable, accessible, timely and implementable manner.

Through investments, ongoing collaborations, and partnerships, we work hand-in-hand in-country to provide lifesaving COVID-19 public health assistance, turning vaccines into vaccinations, training healthcare and public health workers, and strengthening critical health capabilities to better prepare us and the world for future health threats. To be successful, we are leveraging ongoing relationships and building upon trusted networks and partnerships to help countries assess their preparedness and readiness for future outbreaks and pandemics, as well as the sustainability of programs. At the same time, CDC is evaluating its own response readiness and is training and preparing the public health workforce for the future. In that work, CDC’s mission for health equity is a core feature of our public health actions, both in the United States and around the world (*19*). Global health security requires equity; no community, district, or province will be truly healthy until all are.

No nation, including the United States, will be truly safe until all nations have the core public health capabilities and health systems in place to protect the groups that have been economically, socially, and historically marginalized. Through partnership, shared goals, and a global commitment, we can learn from our experience with the global COVID-19 pandemic, advancing health equity and building a strong public health system in every country to prevent and protect against the next inevitable global health threat.
